# Basil (*Ocimum basilicum*) Landraces Can Be Used in a Water-Limited Environment

**DOI:** 10.3390/plants12132425

**Published:** 2023-06-23

**Authors:** Iakovos Kalamartzis, Paschalis Papakaloudis, Christos Dordas

**Affiliations:** Laboratory of Agronomy, School of Agriculture, Aristotle University of Thessaloniki, 54124 Thessaloniki, Greece; zisvos@yahoo.gr (I.K.); pasxalis_pap@hotmail.com (P.P.)

**Keywords:** essential oil content, yield, dry weight, leaf area index, CO_2_ assimilation

## Abstract

Basil (*Ocimum basilicum* L.) is a member of the Labiatae family and is one of the most widely consumed aromatic and medicinal plants in many countries due to its numerous properties and uses. The objective of the study was to determine whether landraces are better adapted to water-limited environments compared to commercial cultivars. Irrigation levels and genotypes affected plant height and leaf area index, with 25% and 33% higher values observed under complete irrigation, respectively. Additionally, limited water availability resulted in a 20% reduction in dry matter yield and a 21% reduction in essential oil yield over the three years in all of the genotypes tested, specifically in the lower irrigation treatment (d_40_), compared to the control treatment (d_100_). The landraces that performed the best under limited water supply were Athos white spike (AWS) and Gigas white spike (GWS), indicating their suitability for environments with limited water resources. The results demonstrate that there are landraces that can be utilized in dryland climates with appropriate water management, enabling water conservation and utilization of fields in water-scarce areas for irrigation purposes.

## 1. Introduction

The modern agricultural system used worldwide, based on intensive agriculture, poses a significant threat to biodiversity [1]. The widespread adoption of intensive agriculture practices has resulted in a decrease in the number of species used in agricultural ecosystems, a decline in the use of crop rotation, and the displacement of locally adapted varieties (landraces) [2,3] with a limited number of hybrids and improved varieties [4]. This decline in agrobiodiversity is exemplified by the fact that the majority of the world’s population relies on products derived from just 12 crops, namely wheat (*Triticum aestivum* L.), rice (*Oryza sativa* L.), maize (*Zea mays* L.), and potato (*Solanum tuberosum* L.), which account for 60% of their diet [1]. Furthermore, the consequences of intensive agriculture’s impact on genetic erosion are evident in the dramatic reduction of varieties within important crop species. For example, in Indonesia, 74% of rice varieties originated from a single genotype, while in the USA, 50% of wheat varieties used by farmers were derived from only nine varieties. Similarly, 75% of potatoes were sourced from four varieties, and 50% of soybeans came from just six varieties [5].

The implementation of intensive agriculture has resulted in several adverse consequences, including increased soil salinity and degradation of the plant growth environment [6,7,8,9,10,11,12,13]. This degradation is not solely attributed to intensive agriculture but is also influenced by climate change, such as rising temperatures and alterations in rainfall patterns, which will further amplify the vulnerability of agricultural production in the future [14,15]. In order to address these conditions, it is crucial to utilize varieties that are well adapted to existing and anticipated adverse changes [16].

Landraces, with their historical evolutionary trajectory and adaptation to stress conditions, can play a significant role in the development of improved varieties that require minimal inputs [1]. By incorporating valuable genes from local varieties, enriching agrobiodiversity with improved varieties becomes a part of the solution to enhance both the yield and quality of agricultural production [16,17,18].

There is a significant interest in conducting systematic evaluations of local varieties to facilitate the identification of alleles, which are the genes responsible for controlling desired traits in different ways [19,20,21]. A study [1] has emphasized that local varieties can serve as potential sources of valuable genes for improving yield and adapting to abiotic stresses, thereby enhancing the productivity and stability of cultivated varieties in adverse environments [1]. However, for a genotype to be widely utilized, its adaptation to challenging climatic conditions must be assessed based on performance-related criteria. Specifically, its resilience (ability to withstand adverse conditions) or tolerance (ability to mitigate the negative impacts of adverse conditions) must be evaluated under specific environments [22].

Therefore, when examining genotypes of aromatic plants such as basil, it is necessary to evaluate them using morphological, physiological, and agronomical characteristics associated with dry biomass yield and essential oil yield [22]. The availability of well-adapted cultivars for aromatic and medicinal plants, particularly basil, across a wide range of growing conditions is limited, and there have been no recent breeding programs focused on developing new cultivars adapted to abiotic stress. Moreover, the absence of high-yielding and high-quality commercial cultivars has resulted in low-income farmers gradually replacing basil farms with other economically profitable plants.

Although there have been some studies on the activity of essential oils from local basil varieties, research on the available genetic resources of basil and their adaptation to abiotic stress remains limited [23,24,25]. Therefore, it is crucial to investigate landraces in order to identify and document local domestic varieties and assess their adaptability to reduced water availability, particularly during the summer period in Mediterranean conditions, which is significant for basil cultivation [1,22,26,27,28].

Water scarcity is a major issue in modern agriculture, especially in Mediterranean countries. Furthermore, climate change and projected scenarios indicate that water availability will become a severe problem for many countries in the coming years, particularly in the Mediterranean region [29]. The efficient utilization of water resources is essential due to the increasing demand for water in domestic, industrial, and agricultural sectors, as well as the impacts of global warming. Water stress is one of the primary limiting factors for crop production worldwide [29]. The economic losses caused by water stress in most crops are substantial, and improved water management can contribute to water conservation and more efficient utilization. This can be achieved by utilizing species and cultivars that are more tolerant to water stress and by applying water during periods of maximum productivity demand [30]. In basil cultivation, the appropriate use of cultivars that are resistant to water stress and can perform better under moderate water stress conditions can be advantageous [20,21,26,27,31]. Additionally, a study [32] demonstrated that there are landraces well adapted to drought that can be utilized in breeding programs to enhance the drought tolerance of maize. Similar results have been reported for other crop species [21,31]. However, there are no studies showing the specific effects of limited water availability on basil landraces. Therefore, the hypothesis tested in this study was whether landraces are better adapted to water-limited environments and exhibit superior adaptation compared to commercial cultivars under conditions of reduced water availability.

The objective of the present study was to determine whether landraces are better adapted to water-limited environments compared to commercial cultivars using morphological, physiological, and agronomical characteristics.

## 2. Results

### 2.1. Plant Height

Plant height was influenced by irrigation level, growth stage, and genotype (Table 1). Both landraces and commercial cultivars showed an increase in plant height from the first to the third measurement (Table 1). Among the genotypes, the tallest variety was Kassandra red spike (KRS), followed by Sweet (a commercial cultivar), and then by Pink spike (PS) cultivar. The shortest variety was Corymb white, followed by the landrace Gigas white spike (Table 1). Significant differences were observed between the two irrigation levels, with the tallest plants found at d_100_, which was by an average 25% higher than at d_40_ across all genotypes and growth stages over the three years of the experiment (Table 1). The greatest difference was observed in KRS and the commercial cultivar during the third year of the experiment, where the difference between the d_40_ and d_100_ treatments ranged from 15% to 102%. PS exhibited the least response to reduced water availability compared to other genotypes.

### 2.2. Leaf Area Index

The leaf area index (LAI) was found to be influenced by growth stages, irrigation level, and genotype (Table 2). The LAI increased from the first to the second measurement in all treatments and genotypes, but it showed a decrease from the second to the third measurement in most cases. Among the genotypes, Sweet, Genovese, and KRS had the highest LAI values, as indicated in Table 2. Across the majority of genotypes, there were significant differences in LAI between the two irrigation levels, with the highest LAI observed at d_100_, averaging a 33% increase. During the second year, the differences between the two irrigation treatments were smaller, whereas in the third year, the differences were more pronounced in the d_100_ treatment. The availability of water had a significant impact on LAI, with limited water resulting in lower LAI in most genotypes, particularly in GWS. Comparing the reduced irrigation treatment to the control treatment, the LAI was considerably lower, as shown in Table 2. The landrace CW did not show any significant changes in LAI.

### 2.3. Chlorophyll Content

The chlorophyll content was observed to be lower in the control treatments compared to the water-limited treatments. Specifically, the CW landrace exhibited a significantly higher decrease in chlorophyll content under the water-limited treatments compared to the other genotypes, as presented in Table 3. On the other hand, certain genotypes such as Athos White Spike (AWS) and Genovese (G) demonstrated an increase in chlorophyll content under the control treatments when compared to the d_40_ treatment. However, overall, there was not a substantial difference observed between the two irrigation levels throughout the three years of the experiments.

### 2.4. Chlorophyll Fluorescence

The quantum efficiency of photosystem (PS) II was found to be influenced by the growth stage, growing season, genotype, and irrigation treatments. Variations were observed among the different genotypes under the same treatment, and in some cases, differences were also observed between the growth stages and irrigation treatments, as presented in Table 4. The most significant difference was observed in the GWS landrace, where there was a 22% variation between the two irrigation treatments in the third year. No significant differences were observed in the other genotypes. Overall, there was a 6% increase in the quantum efficiency of PS II in the d_100_ treatment compared to the d_40_ treatment during the third year.

### 2.5. Assimilation Rate CO_2_

The assimilation rate (A) was found to be affected by the growth stages, irrigation level, and genotype, as presented in Table 5. The PS and Sweet genotypes exhibited the highest A values, as indicated in Table 5. In certain genotypes, the assimilation rate increased during the d_100_ irrigation treatment and declined during the d_40_ treatment. Overall, over the three-year study period, the assimilation rate was 15% higher in the d_100_ treatment compared to the d_40_ treatment. Additionally, in most cases, A was higher during the first growth stage and lower during the second and third growth stages. The greatest difference between the d_100_ and d_40_ treatments was observed during the second growth stage, where it reached 19%.

### 2.6. Dry Weight

Dry weight was observed to be influenced by genotype, irrigation treatments, growth stage, and year, with values ranging from 87 to 468 g/m^2^, as presented in Table 6. Additionally, there was an increase in dry weight for most of the genotypes tested over the three-year period, specifically from the initiation of flowering to full bloom. Overall, the dry weight was higher in most genotypes during the year 2017 compared to the other two years. Furthermore, variations were observed among the different genotypes between the two irrigation treatments. Conversely, in 2018, the dry weight of most cultivars was higher under reduced irrigation, and it was not significantly different between the two treatments. For the landrace Corymb violet (CV), there was an increase in dry weight from the first stage to the second, followed by a decrease from the second to the third stage. In 2020, for the landrace Athos white spike (AWS), there was also an increase in dry weight between the first and second growth stages, but no significant difference was observed between the second and third growth stages. Overall, there was a 20% increase in dry weight in the d_100_ treatment compared to the d_40_ treatment over the three growing seasons.

### 2.7. Essential Oil Content

One crucial quality characteristic of aromatic and medicinal plants is the essential oil content, which was observed to be influenced by growth stages, year, genotype, and their interactions, as depicted in Table 7. Among the genotypes, Corymb White (CW), Pink Spike (PS), and Kassandra Red Spike (KRS) exhibited the highest essential oil content, whereas the Genovese (G) cultivar had the lowest content (Table 7). In most genotypes, there was an increase in essential oil content with irrigation, as it was higher in the d_100_ treatment compared to the d_40_ treatment.

### 2.8. Essential Oil Yield (mL/m^2^)

The essential oil yield of aromatic and medicinal plants, an important yield component, was observed to be influenced by growth stages, year, irrigation level, genotype, and their interactions, as presented in Table 8. The essential oil yield was found to be 21% higher in the d_100_ treatment compared to the d_40_ treatment, attributed to the higher biomass under this treatment. Significant variations in essential oil yield were also observed among the different genotypes tested, with Gigas white spike (GWS) exhibiting the highest yield due to its higher biomass, while the Corymb White (CW) and Corymb violet (CV) genotypes exhibited the lowest yield.

### 2.9. Water Use Efficiency

The water use efficiency (WUE) of the plants exhibited variations based on the growth stage, genotype, and irrigation level, as indicated in Table 9. Generally, WUE was higher during the first growth stage and decreased as the plants grew and produced more biomass. Differences were observed among the different landraces as well as between the landraces and the commercial cultivars that were tested. WUE was generally higher in the reduced irrigation treatment compared to the d_100_ irrigation treatment. This is because the relationship between water supply and biomass production is not linear, and providing more water can lead to a slower increase in biomass production. The genotypes with the highest WUE under reduced irrigation were Corymb violet (CV) and Genovese, while the landrace with the lowest WUE was Corymb White (CW) (Table 9).

### 2.10. Principal Component Analysis (PCA)

The principal component analysis (PCA) was conducted separately for each year of experimentation, considering all cultivars tested and the mean values of the measured parameters under the two different water treatments (d_40_ and d_100_). This analysis provides insights into the relationship between different plant parameters and their contribution to the overall variability observed in the data. The first component represents a set of correlated characteristics related to plant growth and productivity, while the second component captures the variation in physiological traits associated with photosynthetic efficiency and water use efficiency.

**Figure 1:**
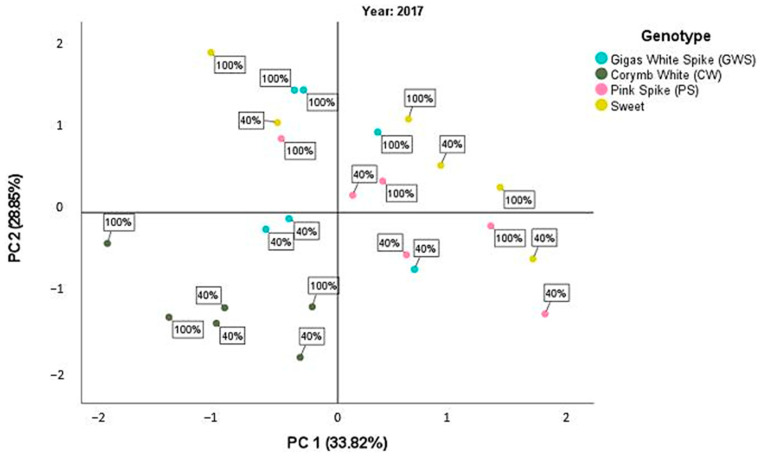
Scatterplot of the PCA score for the first year, 2017, between the different basil cultivars and the two irrigation treatments d_40_ and d_100_.

More specifically, in the first year (2017), the PCA identified two major components with eigenvalues greater than 1, which together explained 62.67% of the total variability in the data. The first component accounted for 33.82% of the variability, while the second component explained 28.85% of the variability (Figure 1). In terms of plant parameters, plant height, leaf area index, dry weight, and essential oil yield showed a positive correlation with the first component. On the other hand, essential oil content exhibited a significantly negative correlation with the first component. The second component showed a positive correlation with physiological plant characteristics, including chlorophyll content, chlorophyll fluorescence, assimilation rate of CO_2_, and water use efficiency (Table 10).

**Figure 2:**
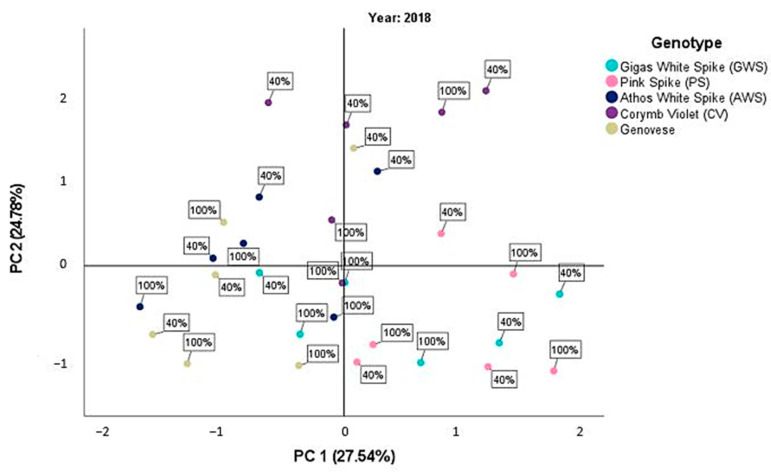
Scatterplot of the PCA score for the second year, 2018, between the different basil cultivars and the two irrigation treatments d_40_ and d_100_.

In the second year (2018), the PCA revealed two major components with eigenvalues higher than 1, which accounted for a total variability of 52.32% in the data. The first component explained 27.54% of the variability, while the second component explained 24.78% of the variability (Figure 2). The correlation patterns observed between the variables in the second year were similar to those observed in the first year. The first component showed positive correlations with morphological parameters such as plant height, leaf area index, dry weight, and essential oil yield. The second component exhibited positive correlations with physiological parameters such as chlorophyll content and chlorophyll fluorescence (Table 10).

**Figure 3:**
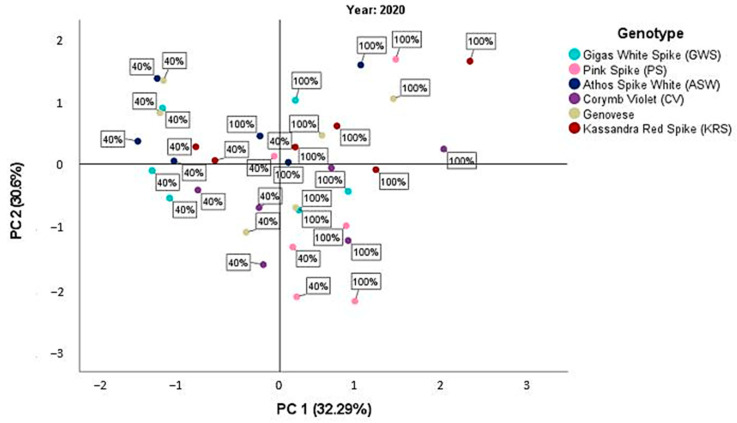
Scatterplot of the PCA score for the third year, 2020, between the different basil cultivars and the two irrigation treatments d_40_ and d_100_.

Similarly, in the third year (2020), the PCA identified two major components with eigenvalues higher than 1, explaining a total variability of 62.89% in the data. The first component accounted for 32.29% of the variability, while the second component explained 30.6% of the variability (Figure 3). The correlation patterns between variables in the third year were also consistent with those observed in the first and second year. The first component showed positive correlations with morphological parameters (plant height, leaf area index, dry weight, and essential oil yield), while the second component exhibited positive correlations with physiological parameters, especially with chlorophyll content and chlorophyll fluorescence (Table 10).

Based on the scatterplots shown in Figure 1 for the first year, it can be observed that Pink Spike and Sweet cultivars exhibited high tolerance to water deficit treatment, as indicated by their high values, particularly along the first component related to morphological parameters and yield. Conversely, Corymb White showed low mean values on both axes, indicating its high susceptibility to water stress. Gigas White Spike displayed a wide range of values, particularly along the second component related to physiological characteristics. Notably, in the d_40_ treatment, Gigas White Spike had negative mean values on the second component, while in the d_100_ treatment, positive mean values were observed.

In the second year (Figure 2), there was greater dispersion between the values of the two irrigation treatments and the genotypes used, particularly across the two components of PCA. Corymb Violet exhibited higher mean values for both water treatments and all measured plant characteristics. Genovese and Gigas White Spike, on the other hand, displayed negative mean values, especially along the second component representing physiological characteristics.

During the third year (Figure 3), although the mean values were closer to the origin of the axes compared to the other two years, all genotypes were negatively affected by water deficit, particularly in terms of morphological characteristics such as plant height and leaf area index. Athos White Spike, Corymb Violet, and Gigas White Spike showed the most significant reduction under water-stressed conditions, with negative mean values along both components. Kassandra Red Spike exhibited positive values, especially along the second component representing physiological characteristics. Pink Spike displayed positive values along the first component related to morphological parameters, even under d_40_ irrigation treatment. Genovese exhibited high dispersion along the axes, sometimes showing positive and sometimes negative values.

## 3. Discussion

To address the impact of climate change, sustainable intensification of agricultural production can be employed, focusing on the efficient utilization of water resources and the development of cultivars or utilization of genotypes that are well-suited to changing environmental conditions. This approach becomes particularly crucial in regions with low rainfall, such as the Mediterranean area, where water stress is a significant environmental factor [33]. Additionally, approximately 18% of the global land surface is situated in semi-arid areas characterized by limited water availability, unpredictable rainfall patterns, land degradation, and limited access to modern agricultural technologies. In such regions, research on crop species and cultivars that are resilient and adapted to these challenging environments becomes a critical option [34].

### 3.1. Plant Height

The plant height of basil has been demonstrated to be influenced by soil water availability [26]. In this study, both basil landraces and the commercial cultivars tested exhibited lower plant height when subjected to low water availability. The factors investigated in the present study included genotypes, growth stages, and the year of experimentation. The environmental conditions, as revealed in this study, can impact the plant height of basil plants. Furthermore, significant differences were observed among the three years of experimentation, which could be attributed to variations in weather conditions. Specifically, higher mean temperatures and lower rainfall in certain years, particularly in June and July, may have affected the growth of basil plants [26,27]. This effect was also noted in the evaluation of five commercial cultivars over a two-year period [26,27].

Overall, the plants exhibited a shorter height, on average by 25%, under the d_40_ irrigation treatment compared to the control treatment. Similar observations have been reported in other studies involving aromatic and medicinal plants, including basil, using commercial cultivars [26,27,35,36,37,38,39]. However, the magnitude of this effect can vary depending on the genotype, as evidenced in the present study. The plant height that was found in this study was higher than in other studies involving basil landraces [38,40], which could be attributed to better growth conditions and the inclusion of genotypes with higher growth potential.

### 3.2. Leaf Area Index

Leaf area is a crucial plant characteristic, particularly for plant species where leaves are the primary harvested plant part, such as many aromatic and medicinal plants [26,41]. Limited water availability has been shown to decrease leaf area, consequently leading to a reduction in photosynthesis, dry matter production, and essential oil production [26]. In this study, the leaf area index was reduced by an average of 33% under conditions of low water availability, reaching up to 91% in certain genotypes, such as Pink Spike (PS) during the first year and at the first measurement. Additionally, the growth of the plants and the leaf area index were influenced by weather conditions, resulting in variations between the three years of the experiment. Similar findings were reported in studies involving commercial cultivars, where the most significant reduction in leaf area index occurred under the lowest water treatment. It has also been documented that water availability, environmental conditions, genotype, and growth stages collectively impact the leaf area of basil plants [27].

### 3.3. Chlorophyll Content

Chlorophyll meters, such as the SPAD meter, have been utilized in various studies to assess the impact of abiotic stresses such as nitrogen deficiency and water stress on plant growth and yield [26,27,42]. In the case of basil, the measurement of chlorophyll content under different water availability conditions revealed that it is not influenced by water availability. Therefore, chlorophyll content cannot serve as a reliable criterion for identifying water stress-tolerant genotypes in basil, unlike in some other plant species [43].

Furthermore, chlorophyll meters are employed to determine leaf senescence and investigate how both abiotic and biotic stresses can affect leaf longevity and the production of assimilates [41,42]. These meters provide a means to nondestructively quantify the level of chlorophyll in leaves under a wide range of growth conditions, which is indicative of their photosynthetic capacity and overall physiological status. By monitoring chlorophyll levels, researchers can gain insights into the impact of stress conditions on leaf function and assess the efficiency of assimilate production in plants [41,42].

### 3.4. Chlorophyll Fluorescence

Chlorophyll fluorescence is commonly used to assess the impact of biotic and abiotic stress on PSII photochemistry, particularly the maximum quantum efficiency, in various plant species, including basil [26,27,43,44,45,46]. The values reported in this study align with findings from other studies conducted on basil and other plant species [27,47,48]. By measuring chlorophyll fluorescence, researchers can evaluate the functionality and performance of the photosynthetic apparatus.

It is worth noting that the photosynthetic electron transport process is generally more resilient to water stress compared to the carbon reduction cycle of photosynthesis. However, under conditions of reduced water availability, there is a decrease in the rate of NADPH generation, which can impact photosynthetic electron transport [44,47]. Furthermore, water stress can lead to an increase in the generation of reactive oxygen species, resulting in photoinhibition and the degradation of key components of the photosynthetic membrane, such as the D1, D2, and CP43 proteins of PSII, as well as pigments and lipids associated with the photosynthetic apparatus [30,49]. These processes can ultimately affect the overall efficiency of photosynthesis in basil and other plant species under water stress conditions.

### 3.5. CO_2_ Assimilation Rate

Water stress affects physiological characteristics of most crop plants [50,51]. However, the effect of water stress on aromatic and medicinal plants and especially on different genotypes and landraces was not determined until recently [26,27]. This is particularly important given the growing interest in alternative crop species such as basil.

The reduction in the CO_2_ assimilation rate (A) observed in some genotypes of basil under water stress ranged from 4% to 113%, which is consistent with findings in other plant species and commercial cultivars of basil [27]. In particular, landraces exhibited an average increase of 14% in CO_2_ assimilation rate under limited water availability, and in some cases, the increase exceeded that of commercial cultivars. When plants experience limited water availability, they tend to close their stomata to conserve water, leading to a reduction in CO_2_ assimilation rate (A) [30,48,49]. Prolonged water stress can further exacerbate this reduction in A.

The use of gas exchange measurements to assess the assimilation rate of CO_2_ in aromatic and medicinal plants, including basil, has gained attention only recently [27]. It is crucial to employ gas exchange techniques to investigate the impact of abiotic and biotic stress on different genotypes, particularly landraces, compared to commercial cultivars in aromatic and medicinal plants. The effect of water stress on photosynthetic efficiency has been studied in several plant species such as faba bean, wheat, and triticale [52,53,54,55]. Expanding this research to include aromatic and medicinal plants will provide valuable insights into their physiological responses to water stress and help identify genotypes that are better adapted to water-limited conditions.

### 3.6. Dry Weight

Dry weight, as an important agronomic characteristic, has been studied in basil and shown to be influenced by genotype, irrigation treatment, and growing season. While the effect of water stress on dry weight has been determined in recent studies on commercial cultivars, its impact on landraces has not been extensively investigated. These studies have revealed that some landraces exhibit tolerance to limited water availability, while others may be more sensitive compared to the tested commercial cultivars [31,56,57,58]. This suggests that certain landraces could be utilized in water-limited environments [31,56,57,58]. It is possible that certain commercial cultivars and landraces have deeper root systems, allowing them to access available water in the soil and meet their water requirements [31,56,57,58]. Tolerant landraces may also possess other mechanisms that enable them to cope with low water availability, such as completing their life cycle before severe water stress occurs or improving water use efficiency mechanisms [21].

Aromatic and medicinal plants have gained increasing interest as alternative crops that contribute to biodiversity enhancement in agricultural systems. They require lower inputs of fertilizers, pesticides, and water compared to conventional crops [1,59]. Furthermore, aromatic and medicinal plants are considered more resilient to climate change [1,59]. Basil, in particular, is a versatile plant species that can be grown for culinary uses as well as for its essential oil content [1,59]. However, the lack of breeding programs for developing new basil cultivars adapted to various environmental conditions and the limited availability of certified seeds have led to a reliance on landraces in basil production. Landraces play an important role in providing farmers with options for cultivating basil in diverse environmental conditions where certified cultivars may be scarce [1,59].

### 3.7. Essential Oil Content and Yield

The essential oil content and yield of basil were found to be influenced by various factors, including genotype, growing conditions, and growth stage [60,61]. In the present study, the irrigation level had a significant impact on the essential oil content, consistent with findings in other plant species [26,27,61,62]. Interestingly, the essential oil content values reported in this study were higher compared to those in previous studies, where the range was around 0.25 mL/100 g [26]. This difference could be attributed to the better growing conditions that existed in the present study.

It is worth noting that water availability tends to affect dry matter yield more significantly than essential oil content, as observed in this study and reported in other studies as well [26]. The essential oil content ranged from 0.50 to 1.94 mL/100 g, showing considerable variation among the three years of the experiment and the tested genotypes. Landraces, in particular, exhibit greater variation compared to commercial cultivars, which can result in higher or lower essential oil content under different treatment conditions.

Overall, the essential oil content of basil is influenced by multiple factors, and the variation observed among genotypes and growing conditions highlights the complexity of this trait [26]. Further studies are needed to elucidate the specific mechanisms and interactions that contribute to the variability in essential oil content in basil.

### 3.8. Water Use Efficiency

In the present study conducted on basil, it was observed that the Genovese cultivar and Corymb violet had the highest water use efficiency (WUE), while Corymb white had the lowest WUE. These findings align with the present study, where higher WUE was observed in lower irrigation treatments [26]. WUE in basil is influenced by factors such as irrigation level, year of experimentation, growth stage, and genotype. Identifying genotypes that are tolerant to water stress and can efficiently utilize water resources is crucial, particularly under limited water supply conditions [26,27,43,63,64,65,66,67,68].

The reported values for WUE in the present study were higher compared to those in other studies. This could be attributed to the cultivation of basil for its leaves, which are harvested at full bloom [63,64,65]. This practice results in lower water consumption, allowing for the conservation of water resources for other crops and the environment [63,64,65,68]. Additionally, previous studies have shown that there is a plateau value in the relationship between water supply and crop production in basil, suggesting that further increases in water supply do not significantly impact dry matter yield [26,27].

Overall, understanding the WUE of basil and its relationship with water supply is important for efficient water management and crop production. Identifying genotypes that exhibit high WUE can contribute to sustainable agricultural practices and the conservation of water resources.

### 3.9. Principal Component Analysis (PCA)

Principal component analysis has been used in different studies to investigate the interrelationships between various traits of plant species [69,70,71]. In the present study, it was found that different genotypes exhibited increased tolerance to limited water availability. Similar results have been reported for other crop species, such as chickpea, where seed yield showed a positive correlation with morphological and agronomic characteristics [70,72]. Furthermore, PCA was employed to provide insights into the genetic diversity based on different traits [70,73]. Several researchers have utilized PCA analysis to classify genotypes and evaluate their response to biotic and abiotic stress [69,70,72,73].

The findings of the present study indicate that consistent relationships existed between the measured variables and their contribution to the overall variability in the data across different years of experimentation. The first component captured variation related to morphological characteristics and productivity, while the second component reflected variation in physiological parameters associated with chlorophyll-related traits. Additionally, the observations of different genotypes under two the irrigation levels highlighted the variability in response to water stress among the genotypes and underscored the varying levels of tolerance or susceptibility to limited water availability throughout the three years of the experiment. Similar differences among growing seasons have also been observed in other crop species [69,73,74,75,76].

## 4. Materials and Methods

### 4.1. Experimental Site and Crop Management

A 3 year (2017, 2018 and 2020) field experiment was conducted in the University Farm of the School of Agriculture of the Aristotle University of Thessaloniki, which is located in Thermi (40°32′9″ N 22°59′18″ E, 0 m)., in Greece. The same field was used over the 3 years, and the soil was clay loam with pH (1:1 H_2_O) 7.77; EC (dS m^−1^) was 1.07, organic matter concentration was 12.40 g kg^−1^, with 56 kg ha^−1^ of N-NO_3_, 24 kg ha^−1^ of P (Olsen), and 204 kg ha^−1^ of exchangeable K and CaCO_3_ concentration of 1.13 g kg^−1^. The soil characteristics were determined according to methods detailed by Sparks et al. [77]. The field was fertilized with 100 and 50 kg ha^−1^ of N and P, respectively, using ammonium phosphate fertilizer before planting. Weather information was recorded during the growth period with an automatic weather station that was close to the experimental site and reported as mean monthly data for all 3 years.

The weather conditions during the 3 years of the experiment varied considerably. In 2017, there was higher rainfall during June but lower rainfall during July and August compared to the 30-year average for the same period. In contrast, 2018 had lower rainfall during the summer months (June, July, and August) compared to both 2017 and the 30-year average. During the third year, 2020, rainfall was low in June and July but higher in August (Table 11). The mean, minimum, and maximum temperatures were higher during all three years of the experiment compared to the 30-year average.

### 4.2. Plant Cultivars Used in the Study

Twenty landraces were evaluated under field conditions during 2015 and 2016 for their agronomic characteristics and also for their essential oil content and essential oil yield. From the 20 basil landraces, different basil landraces were used in this study and had differences in earliness, biomass production, and essential oil content. The landraces that were tested are shown in Table 12.

### 4.3. Crop Management and Experimental Design

The experimental design that was used was a randomized complete block design (RCBD) in a split-split plot arrangement. The two irrigation treatments were the main plots, the genotypes were the sub-plots, and the repeated measures on the three different growth stages were the sub-sub plots with four replications (blocks) per treatment combination. In addition, there were two strips that corresponded to two irrigation treatments of d_100_ and d_40_, and in each strip the genotypes (land races and commercial cultivars) were randomized. Each plot had four rows 5 m long, and each row was 50 cm apart. Basil seeds were sown in a mixture of peat and perlite (9:1) on 4 April 2017, 19 March 2018 and 26 March 2020, and when the basil seedlings were 10 cm in plant height, they were hand-planted to the field on 16 May 2017, 25 April 2018 and 11 May 2020 at a rate of 8 plants m^−2^. Weeds were controlled with hand weeding or tilling when it was necessary. Pest control was achieved with one application of Deltamethrin at a rate of 500 mL ha^−1^.

The different irrigation treatments were applied as described before [26,27], where there were two treatments of 100% and 40% of the net irrigation requirements (IR_n_), and are presented as d_100_ and d_40_, respectively. IR_n_ was calculated from the equation:

IR_n_ = ET_c_ − P_e_ − CR + D_p_ + R_off_ (1)

The equation was used before to calculate the amount of water that had to be applied. Therefore, ET_c_ was the crop evapotranspiration, while the effective rainfall was given as P_e_ and was taken into account only when it was higher than 4 mm on any day, and entire rainfall was considered as effective rainfall, CR was the capillary rise from the groundwater table, D_p_ was the deep percolation, and R_off_ was the runoff. In this study, the CR, D_p_ and R_off_ were negligible because (a) there was no shallow water table problem in the experimental area; thus, the CR value was assumed to be zero; (b) D_p_ was not assumed since the amount of irrigation water was equal to the deficit amount in the root zone; and (c) irrigation was performed with drip irrigation, and there was no runoff.

Reference evapotranspiration (ET_o_) was based on the FAO Penman–Monteith method [78] and was calculated with the following equation:

ET_o_ = [0.408Δ(R_n_-G) + γ[900/(T + 273)]u_2_(e_s_ − e_a_)]/[Δ + γ(1 + 0.34u_2_)] (2)

where ET_o_ is the reference evapotranspiration (mm day^−1^), R_n_ is net radiation at the crop surface (MJ m^−2^ day^−1^), G is soil heat flux density (MJ m^−2^ day^−1^), T is mean daily air temperature at 2 m height (°C), u_2_ is wind speed at 2 m height (m s^−1^), e_s_ is saturation vapor pressure (kP_a_), e_a_ is actual vapor pressure (kP_a_), e_s_ − e_a_ is the saturation vapor pressure deficit (kP_a_), Δ is the slope vapor pressure curve (kP_a_ °C^−1^), and γ is the psychrometric constant (kP_a_ °C^−1^).

Crop evapotranspiration (ET_c_) was calculated with the following equation:

ETc = kc × ET_ο_ (3)

where k_c_ is the crop coefficient.

The following values of crop coefficient (k_c_) were used: for the beginning of flowering, 0.9, for full bloom, 1.1, and for the end of flowering, 1.0 [79].

When the plants were transplanted, 30 mm of irrigation water was applied for better establishment of young seedlings. Subsequently, the different levels of irrigation were applied about 40 days after transplantation and when the plants were at the vegetative stage. The irrigation date was the same for the two treatments and was determined when the soil moisture was at 70% of field capacity of the full irrigation treatment (d_100_) that was considered adequate for plant growth in all growth stages, as described by Kalamartzis [26,27]. The water amount for the deficit irrigation treatment (d_40_) was determined at 40% of the full irrigation treatment. The water was applied with a drip irrigation system after transplanting, with the drippers spaced at 50 cm intervals; the water supply rate of the drippers was 4 L h^−1^. The drip irrigation lines were placed every other row. Volumetric soil water content (θ) was measured with a PR2-type profile probe (Delta-T Devices Ltd., Cambridge, UK) and was used to check the soil water content and to confirm the different water levels. The same irrigation system was extensively used in other experiments [26,27,48].

### 4.4. Sampling

During the growth of the crop, three samplings were conducted at different growth stages covering an area of 1 m^2^. The first sampling was at the beginning of flowering, the second at full bloom, and the third at the end of flowering, starting from the first week of July until the first week of August in all years. The following characteristics were determined: agronomical, morphological, and physiological, water use efficiency (WUE) and essential oil content.

### 4.5. Morphological Characteristics

Two morphological characteristics were determined three times during the growth period of the basil plants for the 3 years that the study lasted. Plant height and leaf area index (LAI) are important characteristics that were determined three times before each sampling by measuring the height of five randomly selected plants per plot from the soil to the top of the plant and obtaining an average value for each plot. LAI was recorded three times before each sampling using the AccuPAR system (model LP-80, PAR/LAI Ceptometer, Decagon Devices, Inc., Pullman, WA, USA).

### 4.6. Physiological Parameters

#### 4.6.1. Water Use Efficiency

The water use efficiency (WUE) for the different cultivars and harvests was determined by dividing the dry weight yield by the total water (rainfall and irrigation) that each treatment received [26,80].

#### 4.6.2. Gas Exchange Measurements

Gas exchange parameters were determined with a portable photosynthesis system (LCi-SD, ADC BioScientific Ltd., Herts, UK) equipped with a square (6.25 cm^2^) chamber. This device was used for measuring CO_2_ assimilation rate (A), transpiration rate (E), stomatal conductance to water vapor (g_s_), and intercellular CO_2_ concentration (C_i_) at the beginning of flowering, full bloom and end of flowering. Measurements were performed on six plants from each plot from 09:00–12:00 in the morning to avoid high vapor-pressure deficits and photoinhibition at midday. Instantaneous water use efficiency (WUE) was obtained by dividing A by stomatal conductance (g_s_) [81].

#### 4.6.3. Chlorophyll Fluorescence

The minimum Chl fluorescence (F_0_) and the maximum Chl fluorescence (F_m_) were measured also in situ with the portable Z995 FluorPen PAR (Qubit Biology Inc. Kingston, ON, Canada). For each plot, 10 young fully expanded leaves were used before each sampling. The maximum quantum efficiency of photosystem (PS) II was calculated as F_v_/F_m_ (F_v_ = F_m_ − F_0_).

#### 4.6.4. Chlorophyll Content

Chlorophyll content readings (SPAD units) were taken with a hand-held dual-wavelength meter (SPAD 502, Chlorophyll meter, Minolta Camera Co., Ltd., Osaka, Japan). This device calculates the leaf chlorophyll content, with maximum absorption at two different wavelengths (400–500 nm and 600–700 nm) and zero absorption in the near-infrared region. However, the device emits radiation in the red and near infrared region (650 nm and 940 nm) and, combining the absorbance at these two wavelengths, calculates the leaf greenness index, giving the final value. For each plot, 10 young fully expanded leaves were used before each sampling. The instrument stored and automatically averaged these readings to generate one reading per plot.

#### 4.6.5. Dry Biomass and Essential Oil Determination

Dry weight yield was determined by conducting three samplings from an area of 1 m^2^; 8 plants per sampling were sampled from the inner row and were randomly selected and cut at the ground level. From each sample, the biomass was weighed to obtain the fresh weight (kg ha^−1^) and allowed to dry at room temperature for a week. When the samples reached a constant weight, they were weighed to obtain the final dry weight. The leaves and flowers were separated from stems by hand and weighed. The essential oil content was determined by using 40 g of dry leaf material subjected to water distillation for 3 h, using a Clevenger apparatus (Sigma, London, UK), and the extracted essential oils were stored at −20 °C. The essential oil content of the plants was determined by a volumetric method (ml 100 g^−1^) [82]. The essential oil yield was determined by multiplying the essential oil content by the dry weight.

### 4.7. Statistical Analysis

The data were analyzed with mixed linear models and the ANOVA method according to the model that involved the effects (main and interactions) of three factors: “irrigation levels” × “genotypes” × “growth stages”. The experiment was implemented according to a randomized complete block design (RCBD) in a split-split-plot design (irrigation levels × genotypes × growth stages), with four replications (blocks) per treatment combination. The irrigation levels were the main plots, genotypes were the sub-plots, and growth stages were the sub-sub plots [83]. A combined ANOVA over the three factors was performed according to the previously described experimental setup. The least significant difference (LSD) criterion was used to test the differences between treatment means, and the significance level of all hypotheses tested was preset at *p* < 0.05. All statistical analyses were performed using the SPSS software package (ver. 17, SPSS Inc., Chicago, IL, USA). The statistical analysis (ANOVA) with SPSS was performed within the frame of mixed linear models using an SPSS syntax code developed and programmed by the authors. Pearson correlations were also applied for all plant characteristics that were measured, and a principal component analysis (PCA) was performed to investigate the similarity between the different irrigation treatments within each cultivar and year of experimentation.

## 5. Conclusions

The present study described the effect of differences in water availability on different landraces and a commercial cultivar in terms of morphological, physiological, and agronomical characteristics and essential oil content of the different genotypes. It was found that the basil genotypes that were tested were not very sensitive to limited water supply; a reduction of water by 60% compared with full irrigation did not significantly affect the dry weight, as it was lower by 34% compared with the full irrigation. Moreover, it was found that there are some landraces that were not affected by the limited water supply and could be used in areas with low water availability and help to conserve precious water resources.

## Figures and Tables

**Table 1:** Combined effect of genotype (A), irrigation (Β) and growth stages (C) on plant height at the two irrigation levels, where 40% and 100% of the net irrigation requirements (IR_n_) are presented as d_40_ and d_100_, respectively, for the three years of experimentation (2017, 2018, and 2020). Data presented are mean values, where LSD is the least significant difference at 0.05 significance level.

Year				Plant Height (cm)			
		Beginning of Flowering		Full Bloom		End of Flowering	
		d_40_	d_100_	d_40_	d_100_	d_40_	d_100_
2017	Gigas white spike (GWS)	26.3 a ^Ϯ^	32.8 a	28.8 b	37.5 a	32.0 a	38.9 a
	Corymb White (CW)	29.2 a	33.8 a	34.2 a	34.8 a	28.6 b	41.6 a
	Pink spike (PS)	41.9 a	47.7 a	43.3 a	49.8 a	48.8 b	56.1 a
	Sweet (S)	52.9 a	56.1 a	55.3 a	58.8 a	57.7 b	67.1 a
LSDA×B×C	7.5						
2018	Athos white spike (AWS)	46.7 a	48.1 a	50.5 a	45.9 a	51.9 a	52.2 a
	Gigas white spike (GWS)	33.2 b	40.0 a	34.3 b	43.0 a	34.8 a	33.5 a
	Corymb violet (CV)	51.2 a	51.0 a	51.5 a	44.6 b	54.1 a	54.9 a
	Pink spike (PS)	43.9 b	51.4 a	48.4 a	46.2 a	40.9 b	50.1 a
	Genovese (G)	45.6 a	46.7 a	49.2 a	48.3 a	50.7 a	51.9 a
LSDA×B×C	5.9						
2020	Athos white spike (AWS)	30.1 b	35.2 a	33.1 b	46.0 a	36.3 b	64.3 a
	Gigas white spike (GWS)	22.5 a	26.0 a	24.8 b	32.0 a	26.8 b	37.0 a
	Corymb violet (CV)	32.6 b	44.0 a	35.1 b	48.8 a	34.7 b	67.8 a
	Pink spike (PS)	32.6 b	41.3 a	33.7 b	46.6 a	41.0 b	64.0 a
	Kassandra red spike (KRS)	31.2 b	42.4 a	31.8 b	54.7 a	37.9 b	76.6 a
	Genovese (G)	32.7 b	39.1 a	33.8 b	47.4 a	38.5 b	63.2 a
LSDA×B×C	5.0						

^Ϯ^ Means in the same genotype followed by the same letter do not differ significantly between the two irrigation treatments d_40_ and d_100_ according to the LSD test (*p* = 0.05).

**Table 2:** Combined effect of genotype (A), irrigation (Β) and growth stages (C) on leaf area index (LAI) at the two irrigation levels, where 40% and 100% of the net irrigation requirements (IR_n_) are presented as d_40_ and d_100_, respectively, for the three years of experimentation (2017, 2018, and 2020). Data presented are mean values, where LSD is the least significant difference at 0.05 significance level.

Year		Leaf Area Index (LAI)					
		Beginning of Flowering		Full Bloom		End of Flowering	
		d_40_	d_100_	d_40_	d_100_	d_40_	d_100_
2017	Gigas white spike (GWS)	1.70 b ^Ϯ^	2.90 a	2.10 b	3.90 a	1.70 b	3.00 a
	Corymb White (CW)	1.20 a	1.50 a	2.00 a	1.60 a	0.60 a	1.00 a
	Pink spike (PS)	1.20 b	2.30 a	1.30 b	2.40 a	2.50 b	3.60 a
	Sweet (S)	2.10 a	2.60 a	1.90 b	3.00 a	2.90 b	4.10 a
LSDA×B×C	1.00						
2018	Athos white spike (AWS)	2.44 a	2.71 a	1.95 a	1.61 a	1.26 a	1.30 a
	Gigas white spike (GWS)	1.82 a	1.53 a	2.01 a	1.48 b	1.77 a	2.12 a
	Corymb violet (CV)	2.43 a	2.19 a	1.93 a	1.77 a	2.30 a	2.08 a
	Pink spike (PS)	0.95 a	1.59 a	1.63 a	1.36 a	1.33 a	1.45 a
	Genovese (G)	3.04 a	2.90 a	2.01 a	1.77 a	1.33 a	1.46 a
LSDA×B×C	0.57						
2020	Athos white spike (AWS)	1.99 a	2.52 a	2.54 a	3.25 a	2.45 b	4.03 a
	Gigas white spike (GWS)	1.29 b	2.49 a	1.82 b	3.05 a	2.06 b	3.00 a
	Corymb violet (CV)	1.70 b	2.66 a	1.88 b	2.93 a	2.27 b	3.84 a
	Pink spike (PS)	2.20 a	1.61 a	2.15 a	2.43 a	2.26 a	3.05 a
	Kassandra red spike (KRS)	2.06 b	3.09 a	2.67 b	3.79 a	2.59 b	4.80 a
	Genovese (G)	2.15 a	2.24 a	2.20 b	3.32 a	2.48 b	4.19 a
LSDA×B×C	0.84						

^Ϯ^ Means in the same genotype followed by the same letter do not differ significantly between the two irrigation treatments d_40_ and d_100_ according to the LSD test (*p* = 0.05).

**Table 3:** Combined effect of genotype (A), irrigation (Β) and growth stages (C) on leaf chlorophyll content at the two irrigation levels, where 40% and 100% of the net irrigation requirements (IR_n_) are presented as d_40_ and d_100_, respectively, for the three years of experimentation (2017, 2018, and 2020). Data presented are mean values, where LSD is the least significant difference at 0.05 significance level.

Year		Chlorophyll Content					
		Beginning of Flowering		Full Bloom		End of Flowering	
		d_40_	d_100_	d_40_	d_100_	d_40_	d_100_
2017	Gigas white spike (GWS)	41.3 a ^Ϯ^	36.9 b	36.9 a	34.5 a	34.0 a	30.7 a
	Corymb White (CW)	40.5 a	36.6 a	40.4 a	32.9 b	29.7 a	22.4 b
	Pink spike (PS)	39.4 a	40.3 a	43.0 a	40.4 a	43.6 a	39.4 a
	Sweet (S)	38.9 a	39.5 a	43.6 a	39.9 a	36.8 a	30.0 b
LSDA×B×C	5.4						
2018	Athos white spike (AWS)	37.0 a	36.5 a	31.6 a	31.6 a	30.5 a	34.9 a
	Gigas white spike (GWS)	37.7 a	35.6 a	37.9 a	36.4 a	42.3 a	35.0 b
	Corymb violet (CV)	45.3 a	43.6 a	46.4 a	41.5 a	42.0 a	45.3 a
	Pink spike (PS)	42.8 a	42.2 a	38.9 a	41.2 a	41.3 a	36.5 a
	Genovese (G)	38.0 a	37.8 a	32.2 a	31.3 a	29.2 a	34.3 a
LSDA×B×C	5.2						
2020	Athos white spike (AWS)	34.8 a	37.0 a	36.0 b	39.0 a	35.4 a	37.4 a
	Gigas white spike (GWS)	41.6 a	42.1 a	40.0 a	41.4 a	34.1 a	36.8 a
	Corymb violet (CV)	44.0 a	43.4 a	41.1 b	44.4 a	42.2 a	43.3 a
	Pink spike (PS)	42.6 a	45.0 a	44.5 a	41.8 a	38.4 b	43.0 a
	Kassandra red spike (KRS)	42.7 b	47.3 a	41.6 a	43.6 a	41.9 a	44.1 a
	Genovese (G)	38.7 a	39.5 a	37.8 b	41.6 a	37.3 a	39.5 a
LSDA×B×C	2.97						

^Ϯ^ Means in the same genotype followed by the same letter do not differ significantly between the two irrigation treatments d_40_ and d_100_ according to the LSD test (*p* = 0.05).

**Table 4:** Combined effect of genotype (A), irrigation (Β) and growth stages (C) on chlorophyll fluorescence at the two irrigation levels, where 40% and 100% of the net irrigation requirements (IR_n_) are presented as d_40_ and d_100_, respectively, for the three years of experimentation (2017, 2018, and 2020). Data presented are mean values, where LSD is the least significant difference at 0.05 significance level.

Year		Chlorophyll Fluorescence					
		Beginning of Flowering		Full Bloom		End of Flowering	
		d_40_	d_100_	d_40_	d_100_	d_40_	d_100_
2017	Gigas white spike (GWS)	0.64 a ^Ϯ^	0.60 a	0.62 a	0.61 a	0.67 a	0.66 a
	Corymb White (CW)	0.58 b	0.64 a	0.56 a	0.56 a	0.62 a	0.57 a
	Pink spike (PS)	0.74 a	0.71 a	0.67 a	0.63 a	0.66 a	0.58 b
	Sweet (S) (C)	0.71 a	0.71 a	0.67 a	0.66 a	0.62 a	0.58 a
LSDA×B×C	0.06						
2018	Athos white spike (AWS)	0.66 a	0.65 a	0.68 a	0.68 a	0.66 a	0.65 a
	Gigas white spike (GWS)	0.72 a	0.71 a	0.71 a	0.67 a	0.69 a	0.70 a
	Corymb violet (CV)	0.68 a	0.67 a	0.72 a	0.70 a	0.72 a	0.68 a
	Pink spike (PS)	0.72 a	0.68 a	0.69 a	0.66 a	0.71 a	0.69 a
	Genovese (G)	0.63 a	0.64 a	0.72 a	0.68 a	0.67 a	0.63 a
LSDA×B×C	0.05						
2020	Athos white spike (AWS)	0.66 b	0.75 a	0.65 a	0.65 a	0.66 b	0.73 a
	Gigas white spike (GWS)	0.66 b	0.74 a	0.62 b	0.76 a	0.70 b	0.75 a
	Corymb violet (CV)	0.69 a	0.71 a	0.67 a	0.68 a	0.71 b	0.76 a
	Pink spike (PS)	0.73 a	0.73 a	0.67 b	0.72 a	0.77 a	0.78 a
	Kassandra red spike (KRS)	0.70 a	0.73 a	0.64 b	0.70 a	0.77 a	0.76 a
	Genovese (G)	0.71 a	0.72 a	0.68 a	0.69 a	0.66 b	0.74 a
LSDA×B×C	0.04						

^Ϯ^ Means in the same genotype followed by the same letter do not differ significantly between the two irrigation treatments d_40_ and d_100_ according to the LSD test (*p* = 0.05).

**Table 5:** Combined effect of genotype (A), irrigation (Β) and growth stages (C) on assimilation rate (A) of CO_2_ at the two irrigation levels, where 40% and 100% of the net irrigation requirements (IR_n_) are presented as d_40_ and d_100_, respectively, for the three years of experimentation (2017, 2018, and 2020). Data presented are mean values, where LSD is the least significant difference at 0.05 significance level.

Year		CO_2_ Assimilation Rate (A)					
		Beginning of Flowering		Full Bloom		End of Flowering	
		d_40_	d_100_	d_40_	d_100_	d_40_	d_100_
2017	Gigas white spike (GWS)	11.2 a ^Ϯ^	13.6 a	8.4 a	11.2 a	9.4 a	9.2 a
	Corymb White (CW)	12.9 a	11.0 a	8.8 a	7.4 a	12.5 a	5.2 b
	Pink spike (PS)	26.7 a	23.5 a	11.0 a	13.8 a	12.3 a	12.4 a
	Sweet (S)	23.1 a	25.5 a	7.0 b	12.9 a	10.1 a	8.6 a
LSDA×B×C	4.8						
2018	Athos white spike (AWS)	9.6 a	5.3 a	6.9 a	5.2 a	8.3 a	11.6 a
	Gigas white spike (GWS)	5.9 a	7.5 a	9.9 a	11.6 a	10.5 a	11.7 a
	Corymb violet (CV)	10.8 a	10.1 a	5.1 a	7.8 a	9.3 a	11.9 a
	Pink spike (PS)	8.1 a	9.6 a	8.3 a	8.6 a	10.2 a	10.7 a
	Genovese (G)	8.2 a	6.6 a	3.7 a	7.9 a	4.3 a	6.7 a
LSDA×B×C	5.0						
2020	Athos white spike (AWS)	4.3 a	4.5 a	3.8 a	4.7 a	5.5 a	3.4 a
	Gigas white spike (GWS)	3.9 a	4.7 a	4.1 a	6.1 a	2.7 a	2.4 a
	Corymb violet (CV)	4.6 b	7.0 a	4.5 a	5.1 a	2.4 a	4.4 a
	Pink spike (PS)	3.3 b	5.8 a	9.6 a	5.0 b	3.2 a	3.4 a
	Kassandra red spike (KRS)	5.0 a	4.3 a	4.6 a	5.3 a	4.7 a	4.1 a
	Genovese (G)	6.0 a	6.0 a	3.4 a	5.2 a	1.8 a	3.4 a
LSDA×B×C	2.3						

^Ϯ^ Means in the same genotype followed by the same letter do not differ significantly between the two irrigation treatments d_40_ and d_100_ according to the LSD test (*p* = 0.05).

**Table 6:** Combined effect of genotype (A), irrigation (Β) and growth stages (C) on dry weight at the two irrigation levels, where 40% and 100% of the net irrigation requirements (IR_n_) are presented as d_40_ and d_100_, respectively, for the three years of experimentation (2017, 2018, and 2020). Data presented are mean values, where LSD is the least significant difference at 0.05 significance level.

Year		Dry Weight (g/m^2^)					
		Beginning of Flowering		Full Bloom		End of Flowering	
		d_40_	d_100_	d_40_	d_100_	d_40_	d_100_
2017	Gigas white spike (GWS)	248 b ^Ϯ^	388 a	248 b	420 a	275 b	455 a
	Corymb White (CW)	107 a	155 a	87 a	131 a	192 a	277 a
	Pink spike (PS)	170 a	228 a	203 a	315 a	280 a	323 a
	Sweet (S)	230 a	260 a	328 b	380 a	390 a	468 a
LSDA×B×C	124						
2018	Athos white spike (AWS)	275 a	233 a	283 a	228 a	265 a	238 a
	Gigas white spike (GWS)	230 a	215 a	218 a	270 a	238 a	200 a
	Corymb violet (CV)	273 a	238 a	310 a	223 b	348 a	353 a
	Pink spike (PS)	170b	258 a	300 a	205 b	198 a	223 a
	Genovese (G)	270 a	233 a	223 a	195 a	213 a	183 a
LSDA×B×C	79						
2020	Athos white spike (AWS)	222 b	290 a	229 a	294 a	372 a	423 a
	Gigas white spike (GWS)	184 a	223 a	220 a	272 a	237 b	304 a
	Corymb violet (CV)	156 b	245 a	270 b	379 a	251 b	343 a
	Pink spike (PS)	119 a	162 a	216 a	206 a	229 b	341 a
	Kassandra red spike (KRS)	241 b	319 a	261 b	340 a	281 b	388 a
	Genovese (G)	183 a	229 a	294 b	379 a	333 a	350 a
LSDA×B×C	67						

^Ϯ^ Means in the same genotype followed by the same letter do not differ significantly between the two irrigation treatments d_40_ and d_100_ according to the LSD test (*p* = 0.05).

**Table 7:** Combined effect of genotype (A), irrigation (Β) and growth stages (C) on essential oil content during 2017, 2018, and 2020, at the two irrigation levels, where 40% and 100% of the net irrigation requirements (IRn) are presented as d_40_ and d_100_, respectively, for the three years of experimentation (2017, 2018, and 2020). Data presented are mean values, where LSD is the least significant difference at 0.05 significance level.

Year		Essential Oil Content (mL/100 g Dry Weight)					
		Beginning of Flowering		Full Bloom		End of Flowering	
		d_40_	d_100_	d_40_	d_100_	d_40_	d_100_
2017	Gigas white spike (GWS)	1.44 a ^Ϯ^	1.63 a	1.28 a	1.22 a	1.16 a	1.35 a
	Corymb White (CW)	1.50 b	1.94 a	1.53 a	1.60 a	1.31 a	1.19 a
	Pink spike (PS)	1.47 a	1.66 a	1.31 a	1.56 a	1.16 a	1.22 a
	Sweet (S) (C)	1.31 a	1.38 a	1.06 a	1.28 a	0.85 a	0.88 a
LSDA×B×C	0.38						
2018	Athos white spike (AWS)	0.75 a	0.69 a	0.63 a	0.50 a	0.56 a	0.75 a
	Gigas white spike (GWS)	0.50 a	0.56 a	0.94 a	0.44 b	0.75 a	0.69 a
	Corymb violet (CV)	0.56 a	0.56 a	0.44 a	0.69 a	0.75 a	0.56 a
	Pink spike (PS)	0.75 a	1.00 a	0.81 a	0.88 a	0.94 a	1.19 a
	Genovese (G)	0.75 a	0.56 a	0.69 a	0.88 a	0.69 a	0.69 a
LSDA×B×C	0.39						
2020	Athos white spike (AWS)	1.31 a	1.31 a	1.03 a	1.09 a	1.28 a	1.12 a
	Gigas white spike (GWS)	1.40 a	1.18 b	1.31 a	1.18 a	1.43 a	1.25 a
	Corymb violet (CV)	1.06 a	1.00 a	1.00 a	0.96 a	0.81 a	1.09 a
	Pink spike (PS)	1.18 a	0.93 a	0.93 a	1.15 a	1.34 b	1.81 a
	Kassandra red spike (KRS)	1.43 a	1.53 a	1.28 a	1.06 a	1.40 a	1.46 a
	Genovese (G)	1.00 a	1.18 a	1.15 a	1.03 a	1.15 a	1.15 a
LSDA×B×C	0.28						

^Ϯ^ Means in the same genotype followed by the same letter do not differ significantly between the two irrigation treatments d_40_ and d_100_ according to the LSD test (*p* = 0.05).

**Table 8:** Combined effect of genotype (A), irrigation (Β) and growth stages (C) on essential oil yield at the two irrigation levels, where 40% and 100% of the net irrigation requirements (IRn) are presented as d_40_ and d_100_, respectively, for the three years of experimentation (2017, 2018, and 2020). Data presented are mean values, where LSD is the least significant difference at 0.05 significance level.

Year		Essential Oil Yield (mL/m^2^)					
		Beginning of Flowering		Full Bloom		End of Flowering	
		d_40_	d_100_	d_40_	d_100_	d_40_	d_100_
2017	Gigas white spike (GWS)	2.02 b ^Ϯ^	4.35 a	2.17 b	3.55 a	2.17 b	4.31 a
	Corymb White (CW)	1.04 a	1.97 a	0.75 a	1.10 a	1.30 a	1.85 a
	Pink spike (PS)	1.40 a	2.06 a	1.53 b	2.74 a	1.53 a	2.27 a
	Sweet (S) (C)	1.50 a	1.88 a	1.83 a	2.68 a	1.81 a	2.30 a
LSDA×B×C	1.05						
2018	Athos white spike (AWS)	1.06 a	0.83 a	0.91 a	0.53 a	0.70 a	0.75 a
	Gigas white spike (GWS)	0.65 a	0.83 a	1.20 a	0.79 a	1.60 a	0.97 a
	Corymb violet (CV)	0.65 a	0.72 a	0.66 a	0.85 a	1.32 a	0.98 a
	Pink spike (PS)	0.73 b	1.34 a	1.25 a	0.87 a	1.08 a	1.44 a
	Genovese (G)	1.12 a	0.70 a	0.79 a	0.82 a	0.65 a	0.59 a
LSDA×B×C	0.61						
2020	Athos white spike (AWS)	1.05 a	1.21 a	0.92 a	1.04 a	1.58 a	1.61 a
	Gigas white spike (GWS)	0.86 a	0.95 a	1.04 a	1.20 a	1.35 a	1.65 a
	Corymb violet (CV)	0.46 b	0.82 a	0.76 b	1.15 a	0.64 b	1.23 a
	Pink spike (PS)	0.52 a	0.47 a	0.50 a	0.81 a	0.96 a	2.03 a
	Kassandra red spike (KRS)	1.16 a	1.55 a	1.21 a	1.00 a	1.21 b	1.92 a
	Genovese (G)	0.68 a	0.94 a	1.31 a	1.41 a	1.40 a	1.51 a
LSDA×B×C	0.32						

^Ϯ^ Means in the same genotype followed by the same letter do not differ significantly between the two irrigation treatments d_40_ and d_100_ according to the LSD test (*p* = 0.05).

**Table 9:** Combined effect of genotype (A), irrigation (Β) and growth stages (C) on water use efficiency at the two irrigation levels, where 40% and 100% of the net irrigation requirements (IRn) are presented as d_40_ and d_100_, respectively, for the three years of experimentation (2017, 2018, and 2020). Data presented are mean values, where LSD is the least significant difference at 0.05 significance level.

Year		Water Use Efficiency					
		Beginning of Flowering		Full Bloom		End of Flowering	
		d_40_	d_100_	d_40_	d_100_	d_40_	d_100_
2017	Gigas white spike (GWS)	14.6 a ^Ϯ^	11.1 a	2.4 a	3.3 a	2.5 a	3.4 a
	Corymb White (CW)	6.3 a	4.4 a	0.8 a	1.0 a	1.7 a	2.1 a
	Pink spike (PS)	12.2 a	8.5 a	12.0 a	9.0 a	2.7 a	2.6 a
	Sweet (S) (C)	16.5 a	9.7 b	19.4 a	10.9 b	3.8 a	3.7 a
LSDA×B×C	4.1						
2018	Athos white spike (AWS)	21.8 a	12.8 b	20.9 a	11.0 b	17.3 a	10.5 b
	Gigas white spike (GWS)	17.0 a	10.4 b	10.4 a	12.0 a	11.8 a	7.1 a
	Corymb violet (CV)	21.6 a	13.1 b	22.9 a	10.7 b	22.6 a	15.6 b
	Pink spike (PS)	12.6 a	12.4 a	14.3 a	9.1 b	9.8 a	7.9 a
	Genovese (G)	21.4 a	12.8 b	16.5 a	9.4 b	13.8 a	8.1 b
LSDA×B×C	4.7						
2020	Athos white spike (AWS)	29.2 a	23.2 b	27.3 a	18.5 b	36.0 a	21.0 b
	Gigas white spike (GWS)	24.3 a	17.9 b	26.2 a	17.2 b	22.9 a	15.1 b
	Corymb violet (CV)	20.6 a	19.6 a	32.2 a	23.9 b	24.3 a	17.0 b
	Pink spike (PS)	15.6 a	12.9 a	25.7 a	13.0 b	22.1 a	16.9 b
	Kassandra red spike (KRS)	31.7 a	25.6 b	31.1 a	21.5 b	27.1 a	19.3 b
	Genovese (G)	24.1 a	18.3 a	35.0 a	23.9 b	32.2 a	17.4 b
LSDA×B×C	6.0						

^Ϯ^ Means in the same genotype followed by the same letter do not differ significantly between the two irrigation treatments d_40_ and d_100_ according to the LSD test (*p* = 0.05).

**Table 10:** Component factor loadings for measured plant parameters in the PCA, which was performed separately for the two irrigation treatments during three growing seasons (2017, 2018, 2020).

	2017		2018		2020	
Plant Parameters	Component 1	Component 2	Component 1	Component 2	Component 1	Component 2
Plant Height	0.679	−0.200	0.387	−0.256	0.429	0.807
Leaf Area Index	0.871	0.011	0.636	−0.025	0.554	0.718
Chlorophyl Content	−0.129	0.680	0.445	0.547	−0.437	0.587
Chlorophyl Fluorescence	−0.006	0.867	−0.052	0.302	0.127	0.706
Assimilation Rate CO_2_	−0.153	0.799	0.006	0.740	−0.584	0.200
Dry Weight	0.971	−0.025	0.880	0.202	0.775	0.366
Essential Oil Content	−0.552	0.198	−0.434	0.629	0.566	−0.024
Essential Oil Yield	0.733	0.055	0.034	0.879	0.933	0.225
Water Use Efficiency	0.008	0.813	0.872	−0.132	0.278	−0.721

**Table 11:** The main weather parameters (mean relative humidity (RH_mean_), rainfall, maximum (T_max_), minimum (T_min_), and mean (T_mean_) temperature) and reference evapotranspiration (ET_o_), for the three years and their comparison with 30-year averages. The weather data were recorded with an automatic weather station close to the experimental site.

	Year 2017			Year 2018			Year 2020			30-Year Average		
	June	July	August	June	July	August	June	July	August	June	July	August
T_max_ (°C)	29.8	34.3	33.8	32.4	34.5	33.8	29.5	34.0	34.1	30.2	32.5	32.2
T_min_ (°C)	17.1	20.5	20.4	18.7	21.2	20.4	18.9	20.8	19.1	15.9	18.2	18.0
T_mean_ (°C)	23.2	27.5	27.1	25.9	27.8	27.1	24.2	27.4	26.6	24.5	26.7	26.0
RH_mean_ (%)	66.7	62.7	63.9	62.3	58.9	62.1	55.4	52.7	55.0	60	58	62
Rainfall (mm)	96.2	8.2	1.1	15.2	1.2	0.8	25.6	13	74.8	32	31	24
ET_ο_ (mm/d)	4.5	5	4.5	4.8	5	5	4.5	4.9	5	4	5	5

**Table 12:** The different landraces and cultivars that were used in the field experiments during the three growing seasons.

2017	2018	2020
Gigas white spike (GWS) (L) ^Ϯ^	Athos white spike (AWS) (L)	Athos white spike (AWS) (L)
Corymb White (CW) (L)	Gigas white spike (GWS) (L)	Gigas white spike (GWS) (L)
Pink spike (PS) (L)	Corymb violet (CV) (L)	Corymb violet (CV) (L)
Sweet (S) (C)	Pink spike (PS) (L)	Pink spike (PS) (L)
		Kassandra red spike (KRS) (L)
	Genovese (G) (C)	Genovese (G) (C)

^Ϯ^ Where L is landrace, and C commercial cultivar.

## Data Availability

The data presented in this study are available on request from the corresponding author.
